# Sex as a Predictor of Outcomes for Symptomatic Carotid Stenosis: A Comparative Analysis between CAS and CEA

**DOI:** 10.3390/jpm14080830

**Published:** 2024-08-05

**Authors:** Pasqualino Sirignano, Costanza Margheritini, Wassim Mansour, Francesco Aloisi, Carlo Setacci, Francesco Speziale, Eugenio Stabile, Maurizio Taurino, on behalf of the IRONGUARD-2 Study Investigators

**Affiliations:** 1 Vascular and Endovascular Surgery Unit, Sant’Andrea Hospital of Rome, Department of General and Specialistic Surgery, “Sapienza” University of Rome, 00189 Rome, Italy; 2 Vascular and Endovascular Surgery Unit, Sant’Andrea Hospital of Rome, Department of Molecular and Clinical Medicine, “Sapienza” University of Rome, 00189 Rome, Italy; 3 Vascular and Endovascular Surgery Unit, Policlinico Umberto I Hospital of Rome, Department of General and Specialistic Surgery, “Sapienza” University of Rome, 00161 Rome, Italy; 4 Vascular and Endovascuar Surgery Unit, “Le Scotte” Hospital of Siena, Department of Medicine, Surgery and Neuroscience, University of Siena, 53100 Siena, Italy; 5 Division of Cardiology, Cardiovascular Department, San Carlo Regional Hospital, “Federico II” University of Naples, 85100 Potenza, Italy

**Keywords:** symptomatic carotid stenosis, carotid revascularization, carotid endarterectomy, carotid artery stenting, female sex, timing

## Abstract

Purpose: Reporting gender-related outcomes for symptomatic carotid lesion revascularization after both endarterectomy (CEA) and carotid artery stenting (CAS) procedures in an unselected group of patients treated by Italian Vascular Specialists. Material and Methods: A retrospective study was conducted on patients presenting with recently symptomatic carotid stenosis treated by CAS and by CEA. The primary endpoint was the 30 days any stroke occurrence rate; secondary endpoints were technical success, occurrence of transient ischemic attack (TIA), acute myocardial infarction (AMI) and death. Demographic, clinical and procedural data were all noted in order to identify the outcome’s determining factor. Results: A total of 265 patients (193 males and 72 females) were enrolled, and of these 134 (50.5%) underwent CEA and 131 CAS (49.5%). At 30 days, the overall new stroke rate was 3.4% (one fatal), and no TIA, AMI or deaths were observed. Among strokes, seven major and two minor strokes were reported, with six after CEA and three after CAS (*p* = 0.32; OR: 2; CI95%: 0.48–8.17). The timing of revascularization has been found to be slightly associated with new stroke occurrence: seven out nine strokes were observed in patients treated within 14 days from symptom onset (5.5% vs. 1.4%; *p* = 0.08, OR: 3.8, CI95%: 0.77–18.56). Lastly, female patients presented a significantly higher risk of post-operative stroke compared to male patients: 6.9% vs. 2.1% (*p*: 0.05; OR: 3.52; CI95%: 0.91–13.52). Conclusions: Our experience seems to suggest that both CEA and CAS provide safe and effective results in treating patients presenting with symptomatic carotid stenosis. Regardless of the type of revascularization, female sex is an independent risk factor for stroke recurrence after treatment.

## 1. Introduction

Sex is a well-known and universally accepted element independently influencing development, response to treatment and outcomes in most pathological situations, particularly regarding cardiovascular and cerebrovascular diseases [1,2]. However, until today, research and medical care have been centered on male physiology, as large randomized clinical trials (RCTs) have predominantly enrolled men [3].

Specifically for carotid disease, all major RCTs enrolled an underpowered number of female patients, whereas the role of sex as a modifier of treatment outcome in symptomatic patients remains uncertain. It was reported as a surgical risk factor for symptomatic women undergoing carotid endarterectomy (CEA) in post hoc subgroup analyses in previous RCTs [4,5,6,7,8,9,10,11,12,13]. In particular, the European Carotid Surgery Trial (ECST) and North American Symptomatic Carotid Endarterectomy (NASCET) demonstrated a significantly increased risk ratio for perioperative adverse event occurrence in women [11,12]. Conversely, the CREST study (Carotid Revascularization Endarterectomy vs. Stenting Trial) did not demonstrate any increase in periprocedural risks in symptomatic women [5]. Moreover, the timing of carotid surgery after a transient ischemic attack (TIA) or stroke was more impactful in women [4,5,6,7,8,9,10,11,12,13]. Indeed, Howard and colleagues’ paper showed significant differences between RCTs in the magnitude of sex differences in treatment effect, indicating that pooling data from these studies to estimate sex differences may not be valid. They concluded that whether or not sex acts as an effect modifier of CAS-CEA treatment effect in symptomatic patients remains uncertain [4].

More recently, in a VASCUNET study analysis among non-selected symptomatic and asymptomatic patients, no significant sex-related differences were found in peri-operative complication rates after interventions for carotid stenosis [14].

Consequently, with the presence of these conflicting data, guidelines available so far are limited by the lack of data about women included in the RCTs, and all the published documents can only suggest, as a good point of practice, the consideration of sex in determining the risk/benefit ratio for every single patient [15,16,17,18].

All the above considered, despite carotid revascularization techniques providing undoubted benefits to symptomatic patients presenting with transient ischemic attacks (TIA) or strokes, differences in postoperative adverse event occurrence between sexes following intervention remain unclear, as well as the relative benefit of CEA vs. carotid artery stenting (CAS) in men and women [19,20].

The aim of the present study is to evaluate whether gender could be effectively considered as an independent factor influencing the outcomes for symptomatic carotid lesion revascularization after both endarterectomy (CEA) and carotid artery stenting (CAS) procedures in an unselected group of patients treated by Italian Vascular Specialists.

## 2. Materials and Methods

A retrospective study was conducted on patients presenting with symptomatic carotid stenosis treated by CAS (pulled from the IRONGUARD-2 Study [21,22]) and by CEA (single-center experience from the Vascular and Endovascular Surgery Unit, Sant’Andrea Hospital, “Sapienza” University of Rome [23]).

CAS procedures were performed from January 2017 to June 2019 in twenty Italian centers. All the study operators, vascular surgeons, cardiologists, and radiologists would have received proper endovascular training and should perform > 50 CAS procedures per year as per study protocol [21,22]. CEA procedures were performed in a tertiary referral Italian Academic Hospital between January 2010 and June 2020 by the same three staff surgeons [23].

### 2.1. Indications for Treatment

TIA and stroke patients were included in the present analysis in cases of carotid stenosis of the internal carotid artery > 50% according to NASCET criteria [24], with a clear time of symptom onset, NIHSS score < 22, and ischemic hemispheric brain infarct < 1/3 of the middle cerebral artery (MCA) area, regardless of blood–brain barrier disruption, in computed tomographic angiography (CTA) or magnetic resonance imaging (MRI), with the patency of MCA in the detectable portions M1 and M2 [25]. Patients were treated according to clinical status at hospital admission within 1 month from symptom onset [18,19]. Exclusion criteria were the following: evidence or previous (<12 months) intracranial hemorrhage or brain surgery; history of intracerebral aneurysms or arteriovenous malformation; common carotid artery ostial lesions (unless untreated simultaneously with index procedure); occlusion of target vessels; intraluminal thrombosis; previous stented target carotid artery; inability to comply with enrollment and follow-up; history of previous life-threatening contrast media reaction; contraindications to aspirin and clopidogrel; known allergy to nickel or titanium; or uncorrectable bleeding disorders.

The present study conformed to the declaration of Helsinki, and ethical committees were notified. All patients enrolled in the study gave their informed written consent to be submitted for CAS and to be included in the study. For each patient, data were anonymized and collected at periprocedural clinical events and as follow-up data.

The current Italian legislation on observational studies does not request the above-mentioned documents when clinical data are anonymized (Official Gazette of the Italian Republic #76, 31 March 2008).

### 2.2. Preoperative Work-Up

The preprocedural work-up included a complete medical history and physical examination; carotid duplex ultrasound (DUS); arch, supra-aortic vessels, and cranial CTA or MRI; and an independent neurological assessment.

### 2.3. Carotid Endarterectomy

The procedural work-up was performed, as well as for the CAS procedure. CEA was performed under general anesthesia with routine intraoperative brain monitoring by means of cerebral oximetry (INVOS TM 5100C Cerebral/Somatic Oximeter; Medtronic Inc., Santa Rosa, CA, USA) and TCD with continuous ipsilateral MCA flow monitoring, or stump pressure in the absence of a suitable temporal bone window. According to the operators’ preferences, patients’ anatomies, and lesions’ characteristics, CEA was performed either by conventional CEA followed by direct suturing closure or patch angioplasty, Chevalier or VanMaele eversion techniques [26,27], or side-to-end common carotid-to-internal-carotid bypass. The revascularization technique was selected based on operator’s preferences and anatomical characteristics. A carotid shunt (Pruitt-Inahara; LeMaitre Vascular Inc., Burlington, MA, USA) was selectively used in the case of clamping-induced cerebral ischemia (reduction in cerebral oximetry value higher than 30%, reduction in middle cerebral artery flow velocity > 50 cm/s, stump pressure < 30 mmHg) [23].

### 2.4. Carotid Artery Stenting

CAS was carried out using dual-layered carotid stent (InspireMD, Boston, MA, USA) implantation with an embolic protection device in place (EPD), as required in the IRONGUARD-2 protocol. The type of EPD (distal filter or proximal occlusion device) was selected based on operators’ preferences/experiences, patients’ anatomies, and lesions’ characteristics [21]. Procedures were preferentially performed via direct common femoral artery puncture, and radial or ulnar access and direct cervical access were selectively performed according to patients’ anatomies [21]. Pre- and post-dilatation were not routinely performed. Atropine (0.5–1 mg) was given intravenously to most patients just before the post-stenting dilation phase to reduce bradycardia and hypotension potentially associated with carotid dilation. All patients underwent an angiographic examination of the culprit carotid lesion in 2 projections and an angiographic examination of the intracranial circulation in anteroposterior and/or lateral projections. The same angiographic imaging was performed at the end of the procedure to determine whether there was any variation in the intracranial blood flow [21].

### 2.5. Concomitant Therapy

*CEA Patients:* All patients underwent antiplatelet/oral anticoagulant therapy discontinuations and received body-weight-adapted low-molecular-weight heparin (100 IU/kg twice daily). For intraprocedural anticoagulation, unfractionated heparin (70 to 100 IU/kg) was administered to maintain an activated clotting time > 250 s. In the first 48 h after CEA, all patients were maintained under body-weight-adapted low-molecular-weight heparin (100 IU/kg twice daily); thereafter, single-antiplatelet therapy with acetylsalicylic acid (100 mg daily) was started and continued indefinitely.

*CAS Patients:* All patients received dual antiplatelet therapy at a standard dose for at least 2 days prior to the CAS procedure, whenever possible; alternatively, intraprocedural clopidogrel loading was performed. For intraprocedural anticoagulation, unfractionated heparin (70 to 100 IU/kg) was administered to maintain an activated clotting time > 250 s. After the procedure, clopidogrel therapy (75 mg daily) was continued for at least 1 month, while acetylsalicylic acid (100 mg daily) was continued indefinitely.

### 2.6. Follow-Up

At the 30th post-operative day, all treated patients were submitted to physical examination, carotid DUS, and neurological assessment by an independent neurologist.

### 2.7. Endpoints and Definitions

The primary endpoint was the occurrence of any stroke within the first 30 days after surgical or endovascular carotid artery revascularization; secondary endpoints were technical success and 30-day follow-up, the occurrence of transient ischemic attack (TIA), acute myocardial infarction (AMI) and death.

Demographic data, clinical data, anatomical data, plaque characteristics and procedural data were all noted and considered as possible factors determining the outcome. Dyslipidemia is defined as low-density lipoprotein > 70 mg/dL, total cholesterol > 135 mg/dL, or triglyceride > 150 mg/dL [27]; arterial hypertension is defined as having a systolic and/or diastolic blood pressure of >140 mmHg and >90 mmHg, respectively; coronary artery disease (CAD) as stable or unstable angina, ejection fraction < 30%, history of myocardial infarction or congestive heart failure; chronic kidney disease (CKD) as abnormalities of kidney function or structure present for more than 3 months, including an eGFR of less than 60 mL/min/1.73 m^2^ on at least 2 occasions [28]; chronic obstructive pulmonary disease (COPD) as a type of progressive lung disease characterized by long-term respiratory symptoms and airflow limitations, in accordance with [29]; and peripheral arterial disease (PAD) as intermittent claudication or chronic limb-threatening ischemia, according to the American Heart Association [30].

Preoperative anatomical characteristics were evaluated by DUS and computed tomographic angiography CTA. Based on DUS examination, carotid plaques were divided and classified into dominantly hyperechoic, isoechoic, or hypo-anechoic, disomogeneous (non-clearly predominant echogenicity), ulcerated (lesions comprising > 2 craters of >3 mm depth or with poorly defined edges and a hazy appearance), thin fibrous cap (a combination of minimum cap thickness < 0.2 mm and a representative cap thickness < 0.5 mm), and post-CEA restenosis [31,32,33].

Neurological complications were classified as follows: 1. TIA was defined as a new transient episode of neurologic dysfunction caused by focal brain or retinal ischemia without imaging evidence of acute infarction. 2. Minor stroke: defined as a new neurological deficit that has completely resolved in 30 days or has increased the NIHSS by ≤3 points compared with the pre-procedural evaluation. 3. Major stroke: defined as a new neurological deficit that has persisted for >30 days and has increased the NIHSS by ≥4 points compared with the preprocedural evaluation [21,22,23]. Either ischemic or hemorrhagic strokes were considered in the present analysis. Diagnosis was in all case confirmed by brain CTA or MRI according to the neurological evaluation.

Technical success was defined as a successful carotid revascularization in the absence of significant residual stenosis (<30%). Procedural success was defined as a technical success in the absence of cardiac and cerebral adverse events during the hospital stay period [21,22,23]. AMI was defined according to the previously reported standard [34].

### 2.8. Statistical Analysis

Data on demographic characteristics and pre-procedural and procedural details were entered into a prospectively compiled database and analyzed as potentially affecting postprocedural outcomes.

Continuous variables were expressed as mean ± standard deviation (SD) and were compared using a paired or unpaired Student’s *t*-test. Categorical variables were expressed as counts and percentages and compared using Fisher’s exact test or the chi-squared test. The odds ratio was calculated to study the primary endpoint for clinical and procedural variables. A 2-sided value of *p* < 0.05 was considered statistically significant. A multivariate logistic regression analysis that included variables with *p* < 0.1 was performed to identify independent predictors of post-operative stroke occurrence after carotid revascularization. Statistical tests were performed using SPSS 25.0 (IBM Corp, Armonk, NY, USA).

## 3. Results

A total of 265 symptomatic patients (193 males and 72 females) underwent carotid revascularization. Overall, 134 patients (50.5%) underwent CEA and 131 CAS (49.5%). Clinical and demographic characteristics of patients included in the present analysis are reported in Table 1. The mean age was 72.52 ± 8.81 years, and was similar between groups (*p* = 0.93). Patients submitted to CAS were more prone to dyslipidemia (*p* = 0.01; OR: 1.98; CI95%: 1.14–3.43), while smoking history was more common in CEA patients (*p* = 0.05; OR: 0.62; CI95%: 0.38–1.01; Table 1).

Carotid plaque characteristics were no different between the groups (Table 2), with a mean target stenosis of 79.9 ± 12.3% (range 50–99). Of note, five cases of symptomatic restenosis were treated by CAS and none by CEA.

In patients submitted to CEA, patch angioplasty was mostly performed (111; 82.8%) and a shunt was needed in 43.3% of cases. CAS procedures were per-protocol performed by a dual layered carotid stent implantation with an EPD in place, and of these the vast majority were filters (70.2%).

The cumulative 30-day new stroke rate was 3.39%, while the death rate was 0.37%. Nine new strokes (one fatal) were recorded out of 265 treated patients: seven major strokes and two minors. Of them, six strokes (two hemorrhagic) were observed after CEA and three (one fatal hemorrhagic conversion) after CAS (*p* = 0.32; OR: 2; CI95%: 0.48–8.17).

In the whole series, no TIA, AMI, or other deaths were observed at the end of the follow-up period.

The timing of revascularization has been found to be slightly associated with any new stroke occurrence: seven out of the nine new strokes reported were observed in patients treated within 14 days, while two events occurred in procedures performed after 14 days from hospital admission (5.5% vs. 1.4%; *p* = 0.08, OR: 3.8, CI95%: 0.77–18.56).

Dividing the study population by sex, female patients were less frequently affected by comorbidities compared to male patients, but plaque composition (*p* = 0.89) and revascularization timings (*p* = 0.93) were not different between the two groups (Table 3).

At univariate analysis, female sex was the only characteristic associated with a significantly higher risk of suffering a post-operative stroke after symptomatic carotid revascularization compared to male: five new neurological events occurred among 72 (6.9%) women treated versus four in 193 (2.1%) men (*p*: 0.05; OR: 3.52; CI95%: 0.91–13.52). No differences were found in terms of performed revascularization; among five new strokes in female patients, four were observed after CEA and one after CAS (*p*: 0.28; OR: 3.2; CI95%: 0.34–30.5); consistently, two strokes after CAS and two after CEA were observed in men (*p*: 0.94; OR: 1.07; CI95%: 0.14–7.8). The role of female sex in predicting the outcome was also confirmed in the multivariate analysis (Table 4).

## 4. Discussion

The most noticeable point emerging from our retrospective analysis on revascularization outcomes in recently symptomatic patients is that, regardless of the revascularization technique, results in males were highly different from those obtained in female patients. Univariate (*p*: 0.05; OR: 3.52; CI95%: 0.91–13.52) and multivariate (*p* = 0.05; OR: 3.94; CI95%: 0.97–16.02) analyses led to the identification of female sex as an independent predictor of stroke after CEA and CAS, and this divergence occurred in a population with a higher frequency of cardiovascular risk factors in men.

However, these figures form a picture of carotid revascularization, either by CEA or CAS, performed in recently symptomatic patients with an any new stroke 30 day occurrence significantly lower than the 6% threshold indicated by contemporary literature and available guidelines [16,17,18]. Indeed, in our series, even if all procedures were performed in high-volume centers by skilled operators, the cumulative strokes rate was 3.4% without differences between open and endovascular interventions (*p* = 0.32; OR: 2; CI95%: 0.48–8.17).

The latest available ESVS guidelines still suggest CEA as the preferred treatment in patients with symptomatic carotid stenosis; CEA provided significantly better outcomes than CAS in terms of stroke occurrence and death rate [16]. However, available evidence arises from RCTs conducted with first-generation single-layer carotid artery stents, which are more prone to embolic complications and early plaque prolapse compared to newly developed dual-layer stents [35]. Indeed, the routine use of a modern CAS procedure with dual-layered stent implantation may be the cause of the lack of distinctions between CAS and CEA, in present experience. Even in patients with symptomatic or unstable lesions, this type of stent significantly improved CAS results by preventing acute plaque prolapse in the first postoperative period [35]. This could explain our results in a population presenting a significant incidence of vulnerable plaque prone to cerebral embolism during CAS (hypoanechoic 32.8%, disomogeneous 19.8%, ulcerated 9.3%, and thin fibrous cap 4.6%).

In addition to all other technical and demographic characteristics, even in the present series, the timing of revascularization has been found to be a crucial factor in determining the outcome, at close to statistical significance. Seven out of the nine new strokes reported were observed in early-treated cases, while two events occurred after delayed procedure (12.24% vs. 1.4%; *p* = 0.0001, OR: 9.9, CI95%: 2.38–41.15) [23].

Considering again the role of sex in determining outcome as a pillar of our observations, there are many literature findings that need to be clarified. Recently, the VASCUNET study by Venermo et al., which includes over 200.000 procedures, showed no statistically significant sex-related differences in stroke, major cardiac events, or death rates after carotid revascularization, regardless of symptoms at presentation [14]. Even though sex was not associated with complications, age acted as an independent risk in determining the outcome. By focusing on symptomatic patients, Howard et al., in their patient-level meta-analysis, were only able to draw the conclusion that the role of sex, as an effect modifier of the CAS-to-CEA treatment in symptomatic patients, remains unclear, even assuming that each RCT is insufficient to provide a specific answer and considering that no trial has demonstrated any gender-related discrepancy [4]

Looking at RCTS and related meta-analysis, female sex was reported as a surgical risk factor for symptomatic patients undergoing CEA in post hoc subgroup analysis. The ECST and the NASCET demonstrated a significantly increased risk ratio for perioperative adverse event occurrence in women, while the CREST study did not demonstrate any increase in periprocedural risks in symptomatic women [5,6,7,8,9,10,11,12,13]. Consistently, Goicoechea and coworkers found that women with symptomatic carotid stenosis suffered a higher rate of cerebrovascular events and stroke after revascularization when compared to males [20]. Unlike our data, however, the complication rate between males and females was only different after CEA, but not after CAS. Probably, the imbalance in performed procedures (104.421 CEA vs. 2.156 CAS) could have influenced their analysis. On the contrary, in our experience, even though the number of patients was lower, both procedures were equally balanced and represented. Another recent Italian experience on CEA for symptomatic stenosis, recently published by D’Oria et al., showed no sex-related differences in symptomatic patients, with female patients presenting a longer survival rate [36], once again emphasizing the huge uncertainty in this field.

The crucial finding of our study represents an element of uncertainty, like in the entire published literature: although no differences were noticed in terms of symptoms and plaque characteristics, women’s outcomes after carotid revascularization seemed to be worse.

Although the real predictive value of our results is supported by significant statistical difference, the design of the present retrospective study presents several limitations. First, enrolled patients were pulled out from a prospective registry on CAS and a single center surgical experience, with a relatively low number of included cases. Patients were not treated by the same operators and in the same setting, representing a potential enrollment bias. Moreover, neither randomization nor a propensity score were performed. Data-pooled form studies designed for a different aim indubitably lack potentially interesting details such as preoperative Rankin scale values, qualitative plaque analysis (except for DUS evaluation), common and internal carotid artery diameter evaluation, postoperative MRIs and cognitive test assessments. Furthermore, both urgent and emergent patients were considered in the present series, despite the well-known differences between these subsets of patients [23,25]. Lastly, because either CEA or CAS were performed in this experience, patients were not always treated according to currently available guidelines, suggesting CEA as the preferred treatment for symptomatic patients [16,17,18]. However, the same guidelines allow CAS in selected patients, presenting contraindications to CEA [16,17,18], as in the present paper.

In conclusion, our experience seems to suggest that, regardless of the type of revascularization, female sex is an independent risk factor for stroke recurrence after surgical or endovascular treatment in recently symptomatic carotid lesions.

However, we do not believe that this is the time for a “moratorium” on revascularization, either with CEA or CAS, in female patients with recently symptomatic internal carotid stenosis.

From a speculative point of view, we think that our work could represent a further hypothesis generator and a wake-up call for the realization of a randomized study with a male/female ratio of 1:1 to provide a definitive statement on the role of sex in determining outcome after symptomatic carotid revascularization.

## Figures and Tables

**Table 1:** Demographic and clinical characteristics of all patients included in the present series.

	Patients (N = 265)	CEA (N = 134)	CAS (N = 131)	*p*
**Age (years ± SD; range)**	72.52 ± 8.81; 50–93	72.47 ± 8.91; 50–93	72.52 ± 8.97; 50–89	0.93
**Male (n; %)**	193; 72.8	93; 69.4	100; 76.3	0.20
**Hypertension (n; %)**	225; 76.3	113; 84.3	112; 88.5	0.79
**Diabetes (n; %)**	91; 34.3	44; 32.8	47; 35.8	0.60
**Dyslipidemia (n; %)**	190; 71.7	87; 64.9	103; 78.6	0.01
**CAD (n; %)**	89; 33.6	38; 28.3	51; 38.9	0.06
**PAD (n; %)**	38; 14.3	19; 14.1	19; 14.5	0.93
**CKD (n; %)**	34; 12.8	17; 12.7	17; 12.9	0.94
**COPD (n; %)**	67; 25.3	34; 25.4	33; 25.2	0.97
**Smoking History (n; %)**	153; 57.7	85; 63.4	68; 51.9	0.05

CAD: coronary artery disease; PAD: peripheral arterial disease; CKD: chronic kidney disease; COPD: chronic obstructive pulmonary disease.

**Table 2:** Carotid plaque characteristics, and type and timing of performed interventions of all patients enrolled in the present series.

	Patients (N = 265)	CEA (N = 134)	CAS (N = 131)	*p*
**Right-Side Stenosis (n; %)**	115; 43.4	58; 43.3	57; 43.5	0.97
**Target Lesion Stenosis (% ± SD)**	79.90 ± 12.34;	82.43 ± 9.67;	77.31 ± 13.48;	0.47
**Contralateral Stenosis (% ± SD)**	47.58 ± 20.41;	47.08 ± 21.79;	48.09 ± 19.05;	0.83
**Plaque Composition**				
**Hyperechoic (n; %)**	29; 10.9	13; 9.7	16; 12.2	0.08
**Isoechoic (n; %)**	43; 16.2	20; 14.9	23; 17.5	
**Hypoanechoic (n; %)**	84; 31.7	41; 30.6	43; 32.8	
**Disomogeneous (n; %)**	51; 19.2	25; 18.6	26; 19.8	
**Ulcerated (n; %)**	33; 12.4	21; 15.7	12; 9.3	
**Thin Fibrous Cap (n; %)**	20; 7.5	14; 10.6	6; 4.6	
**Post-CEA Restenosis (n; %)**	5; 2.3	-	5; 3.8	
**Performed Intervention**				
**CEA and Direct Suture (n; %)**		4; 3.1	-	NA
**CEA + Patch Angioplasty (n; %)**		111; 82.8	-	
**Eversion CEA (n; %)**		11; 8.2	-	
**CCA-ICA Bypass (n; %)**		8; 5.9	-	
**Shunt Insertion (n; %)**		58; 43.3	-	
**CAS with Distal Filter (n; %)**		-	92; 70.2	
**CAS with Proximal Occlusion (n; %)**		-	39; 29.8	

CEA: carotid endarterectomy; CCA: common carotid artery; ICA: internal carotid artery; CAS: carotid artery stenting. NA: not applicable.

**Table 3:** Demographic and clinical characteristics, carotid plaque characteristics and timing of performed interventions in male and female patients.

	Male (N = 193)	Female (N = 72)	*p*
**Age (years ± SD; range)**	72.86 ± 9.54; 50–93	72.62 ± 9.22; 50–89	0.31
**Hypertension (n; %)**	162; 83.3	63; 87.5	0.04
**Diabetes (n; %)**	64; 33.1	27; 37.5	0.51
**Dyslipidemia (n; %)**	143; 74.1	47; 65.3	0.01
**CAD (n; %)**	72; 23.6	17; 23.6	0.03
**PAD (n; %)**	33; 17.1	5; 6.9	0.03
**CKD (n; %)**	26; 13.5	8; 11.1	0.61
**COPD (n; %)**	55; 28.4	12; 16.6	0.04
**Smoking History (n; %)**	111; 57.5	42; 58.3	0.04
**Plaque Composition**			
**Hyperechoic (n; %)**	19; 9.8	10; 13.8	0.64
**Isoechoic (n; %)**	34; 17.6	9; 12.5	
**Hypoanechoic (n; %)**	62; 32.1	22; 30.5	
**Disomogeneous (n; %)**	35; 18.1	16; 22.2	
**Ulcerated (n; %)**	23; 11.9	10; 13.8	
**Thin Fibrous Cap (n; %)**	17; 8.8	3; 4.1	
**Post-CEA Restenosis (n; %)**	3; 1.7	2; 3.1	
**Timing of Surgery**			
**<48 h (n; %)**	36; 18.6	13; 18.1	0.93
**48 h–14 days (n; %)**	60; 31.1	21; 21.2	
**>14 days (n; %)**	97; 50.3	38; 60.7	

**Table 4:** Multivariate analysis on factors related to post-operative stroke after revascularization in the present series.

Post-Operative Stroke Occurrence after Revascularization			
Included Variables	*p*	OR	CI95%
**Female Sex**	0.05	4.29	0.97 to 19
**CAD**	0.37	0.36	0.04 to 3.35
**COPD**	0.73	1.35	0.23 to 7.72
**Diabetes**	0.19	0.23	0.02 to 2.07
**Timing of Surgery > 14 days**	0.76	0.71	0.06 to 8.27
**ROC curve analysis**			
**Area under the ROC curve (AUC)**	**Standard Error**	**CI95%**	
0.831	0.08	0.78 to 0.87	

CAD: coronary artery disease; COPD: chronic obstructive pulmonary disease.

## Data Availability

The raw data supporting the conclusions of this article will be made available by the authors on request.
